# Nocardiosis in glomerular disease patients with immunosuppressive therapy

**DOI:** 10.1186/s12882-020-02179-9

**Published:** 2020-11-26

**Authors:** Yuzhang Han, Zineng Huang, Huifang Zhang, Liyu He, Lin Sun, Yu Liu, Fuyou Liu, Li Xiao

**Affiliations:** Department of Nephrology, 2nd Xiangya Hospital, Central South University, Changsha, Hunan China

**Keywords:** Nocardiosis, Glomerular disease, Glucocorticoids, Sulfanilamide

## Abstract

**Background:**

Glomerular disease patients have a high risk of infection, which contributes to the progression of disease per se and mortality, especially in those with long-term use of glucocorticoids and (or) immunosuppressive agents. Cases of sporadic nocardiosis have been reported in glomerular disease patients, and this observation was conducted to comprehensively understand the manifestations of and treatments for nocardiosis, which is commonly misdiagnosed as pneumonia or tuberculosis or even as lung cancer or metastatic tumors in glomerular disease patients.

**Methods:**

We reviewed the demographic characteristics, laboratory abnormalities, radiological features, and treatments of 7 patients with nocardiosis and glomerular disease receiving steroids and immunosuppression therapy at the nephrology department of the Second Xiangya Hospital between 2012 and 2019.

**Results:**

It was found that all 7 patients had been receiving methylprednisolone for renal disease at a median dose of 20 mg per day and a median duration of 4 months before developing nocardiosis. There were 4 males and 3 females, and the median age was 52.14 years. All 7 patients had hypoalbuminemia at the time of admission. In addition, various cystic abscesses in the subcutaneous tissue, with or without lung and brain involvement, were observed in these patients. Encouragingly, body temperatures returned to normal, and subcutaneous abscesses diminished or disappeared with compound sulfamethoxazole treatment alone or in combination with linezolid, imipenem and mezlocillin/sulbactam.

**Conclusions:**

It was shown that multisite abscesses, including subcutaneous, pulmonary and cerebral abscesses, were the common manifestations of nocardiosis in glomerular disease patients. Sulfonamide was the first-line antibiotic therapy for nocardiosis, and combinations of other antibiotics were also needed in some serious cases.

## Background

Glomerular diseases are still common causes of end-stage renal disease (ESRD), a global health epidemic affecting more than 2 million people worldwide [1–4]. Many glomerular diseases are immunologically mediated disorders [1, 5–8], and immunosuppressive medications put glomerular disease patients at a high risk of various infections, such as pneumonia, *Mycobacterium tuberculosis* and hepatitis B virus reactivation, which contribute to the progression of disease per se and mortality [9–14]. In recent years, accumulating data have shown an increased rate of opportunistic infections in glomerular disease patients with long-term use of glucocorticoids and (or) immunosuppressive agents, especially in nephrotic syndrome, IgA nephropathy, lupus nephritis and vasculitis patients [14–20].

*Nocardia spp*. are gram-positive bacteria belonging to aerobic actinomycetes, which are ubiquitous in soil [21]. Nocardia are rare in healthy individuals but are isolated with increasing frequency from the clinical materials of immunocompromised patients, which can cause a disseminated infection and eventually lead to life-threatening outcomes [22–24]. Recently, Nocardia infection, also called nocardiosis, received increasing attention from clinical nephrologists. Sporadic case reports have shown that nocardiosis manifests as fever and various tissue nodules, mainly pulmonary and subcutaneous lesions [25]. Serious cases of nocardiosis can lead to disseminated infections and even the emergence of brain abscesses [26–29]. Unfortunately, nocardiosis is quite complex and rare, and it is difficult to differentiate it from other fever-related diseases, lung cancers and metastatic tumors [29–31]. Misdiagnosis and delayed treatment of nocardiosis are common in glomerular disease patients, which has brought a challenge for the comprehensive understanding of nocardiosis.

The value of clinical guidance from previous sporadic cases may be limited by the small number of patients; thus, we reviewed 7 glomerular disease patients with nocardiosis who received steroids and (or) immunosuppressive agents. We focused on the typical manifestations, antibiotic selection, changes in inflammatory biomarker levels and imaging of nocardiosis after treatment.

## Methods

This observation was conducted to review the clinical records of 7 glomerular disease patients affected by nocardiosis at the nephrology department of the Second Xiangya Hospital of Central South University (a tertiary hospital with 3500 beds) between 2012 and 2019. The data are from the department depository, and 9292 glomerular disease patients were followed during this period. The involvement of 2 noncontiguous organs is defined as disseminated nocardiosis. The inclusion criteria were as follows: glomerular disease patients with immunosuppressive therapy; cough or expectoration, unexplained renal deterioration, and subcutaneous, pulmonary or cerebral abscess suggesting nocardiosis; and confirmed nocardiosis through a positive Nocardia culture, acquired from the puncturing of abscesses.

We collected demographic characteristics, including sex and age, as well as renal diseases, laboratory abnormalities, radiological features, and treatments in 7 culture-proven cases. To analyze the therapeutic effect, we analyzed inflammatory biomarkers (N%, percentage of neutrophils; CRP, C- reactive protein; PCT, procalcitonin) and indicators of renal function before and after treatment. For biochemical data, serum albumin was detected by bromocresol green colorimetry, and serum creatinine was measured by the sarcosine oxidase method. For imaging evaluation, B-mode ultrasonography, computed tomography (CT) and magnetic resonance imaging (MRI) were used. For microbiological tests, gram staining and modified acid-fast staining were used for direct bacterioscopy; the streak plate method was used for Nocardia isolation; and blood-containing medium and Lowenstein-Jensen medium were used for culture. Considering the risk factors and antibiotic selections for nocardiosis, we were particularly concerned with the categories, doses, and courses of immunosuppressive agents and antibiotics, respectively.

All statistical analyses were completed using SPSS 20.0, IBM, Chicago, IL, USA. Continuous variables were expressed as the means with standard deviations (SDs), while discrete variables were expressed as the medians with interquartile ranges (IQRs). Paired t-tests were used to compare the inflammatory biomarker levels of patients before and after antibiotic treatment.

## Results

### Basic characteristics of patients infected with Nocardia

Seven glomerular disease patients suffering from nocardiosis were enrolled: 5 had nephrotic syndrome (71.4%), 1 had lupus nephritis, and 1 had IgA nephropathy. Renal histology showed that 3 of 7 patients had membranous nephropathy. The median time between kidney disease diagnosis and nocardiosis was 6 months (IQR, 6–10.5 months). There were 4 males and 3 females, and the median age was 52.14 years (IQR, 50–61 years). Four patients had concomitant diabetes, including 3 glucocorticoid-dependent patients. One patient was even diagnosed with pneumonia 2 months prior, and the other patients had no history of respiratory system diseases (Table 1).

**Table 1:** The basic characteristics of 7 patients affected by Nocardia

Patient	Sex	Age	Smoking history	DM	Renal disease	Renal histology	Baseline eGFR (mL/min/1.73m^2^)	Baseline hematuria	Baseline albumin		Methylprednisolone		Immunosuppressor			Baseline lymphocytes count (10^9^/L)	Time to remission of kidney disease	Time between kidney disease diagnosis and nocardiosis	Other concomitant disease
									serum (g/L)	urine	dose (mg qd)	course	category	dose	course				
1	Male	50–60	20^a^,20 years	–	NS	membranous nephropathy	32.97	+	29.6	3274.34 mg/d	16	6 months	tacrolimus	0.5-1 mg qod, 16.10 ng/mL	6 months	1.83	–	2 years	–
2	Male	20–30	–	SDM	NS	HBV associated glomerulonephritis (membranous nephropathy)	155.5	–	22.5	685.96 mg/d	16	5 months	–	–	–	1.18	–	9 months	Chronic HBV infection
3	Female	60–70	–	–	Lupus nephritis	–	29.79	+	26.1	437.78 mg/d	16	2 months	cytoxan	0.4 g in total	–	0.53	–	1 year	Hypertension; Cerebral infarction
4	Male	60–70	–	DM	NS	–	74.41	–	30.6	–	32	4 months	–	–	–	0.45	4 months	4 months	–
5	Male	50–60	40^a^, 30 years	–	NS	membranous nephropathy	115.25	–	30.2	+	28	4 months	–	–	–	0.87	6 months	6 months	Hypertension Cerebral infarction
6	Female	50–60	–	SDM	NS	–	99.35	+	28.3	+	20	6 months	tacrolimus	1 mg qd, 9.4 ng/mL	2 months	0.67	–	6 months	–
7	Female	50–60	–	SDM	IgA nephropathy	IgA nephropathy (FSGS)	107.26	+++	30.9	+	40	4 months	–	–	–	0.62	–	6 months	Iatrogenic Cushing’s disease

*Abbreviation*: *DM* diabetes mellitus, *eGFR* estimated glomerular filtration rate, *NS* Nephrotic syndrome, *SDM* Steroid diabetes mellitus, *HBV* hepatitis B virus, *FSGS* focal segmental glomerulosclerosis

^a^Numbers of cigarettes per day

### Immunosuppressive treatment before and after Nocardia infection

All 7 patients had been receiving methylprednisolone for renal disease at a median dosage of 20 mg (IQR, 16–32 mg) per day and a median duration of 4 months (IQR, 4–6 months) before developing nocardiosis. Furthermore, 3 patients had been taking a combination of methylprednisolone and an immunosuppressor, including 2 with tacrolimus (*n* = 2) and 1 with cyclophosphamide (Table 1). Six patients developed nocardiosis within 4 months after the initiation of immunosuppressive treatment. The baseline lymphocyte count in 4 patients decreased (Table 1). After Nocardia infection, 3 patients, including the two with disseminated nocardiosis, decreased the dose of immunosuppressive treatment; 1 patient stopped immunosuppressive treatment; and 3 patients did not receive modifications.

### Renal and extrarenal manifestations

**Fig. 1:**
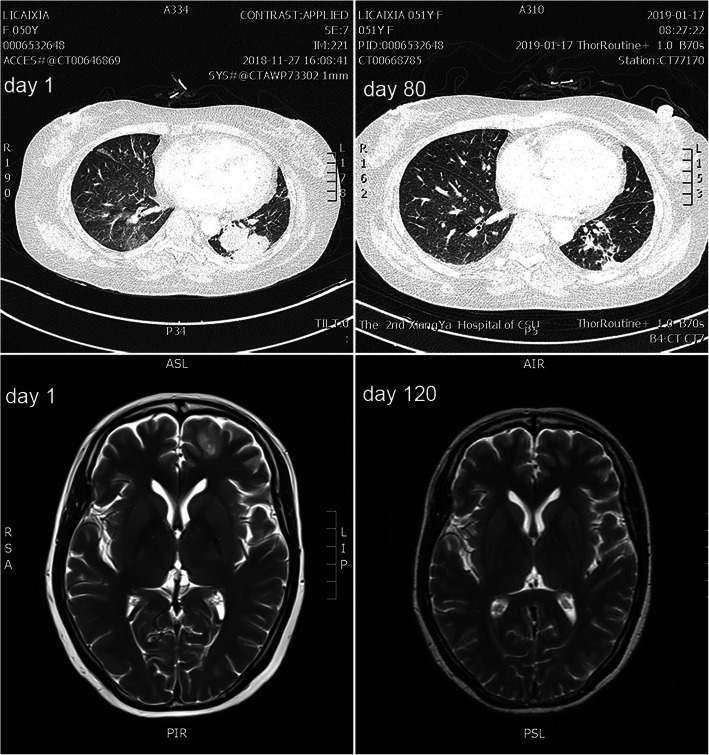
Lung CT and head MRI images of No.7 patient

Before nocardiosis, all 7 patients had already presented with hypoalbuminemia; 6 patients had proteinuria; 4 patients had hematuria; 3 patients exhibited a decreased eGFR; and only 2 patients achieved nephrotic syndrome remission after immunosuppressive treatment, with the disappearance of edema (Table 1). After nocardiosis, all 7 patients exhibited hypoalbuminemia and elevated 24-h urinary protein concentrations at the time of admission, 2 patients exhibited increased serum creatinine, and 1 patient had acute kidney injury (AKI) with a serum creatinine level of 267.5 μmol/L (Table 2). In addition, only 3 patients had fever. Six patients showed respiratory system involvement: 4 with cough, 3 with expectoration, 2 with pleural effusion, 1 with chest distress and 1 with dyspnea (Table 3). Notably, B-mode ultrasonography showed various cystic abscesses in the subcutaneous tissue in all 7 patients, which was accompanied with or without lung and brain involvement on CT or MRI (Fig. 1). All patients underwent head MRI, and 2 patients showed brain abscesses; both of them had no central nervous system symptoms. One patient taking 40 mg qd methylprednisolone for 4 months and the other patient taking a combination of methylprednisolone and tacrolimus presented with serious disseminated disease, characterized by multisite nodules, including subcutaneous nodules, concomitant with pulmonary or cerebral abscess. And abscess was the source of bacteriological diagnosis.

**Table 2:** Comparison of partial inflammatory biomarkers of 7 patients before and after antibiotic treatment

Patient	Before treatment							After treatment^**a**^						
	Inflammatory biomarkers				Renal function			Inflammatory biomarkers				Renal function		
	WBC × 10^9^ /L	N%	CRP mg/L	PCT ng/mL	ALB g/L	Sr μmol/L	24 h UP mg/d	WBC × 10^9^ /L	N%*	CRP* mg/L	PCT* mg/L	ALB g/L	Sr μmol/L	24 h UP mg/d
1	19.7	85	8.39	0.89	20.2	139.3	4793.00	12.8	81.80	2.21	0.32	26.6	138.3	3916.60
2	15.04	86.8	26.60	0.26	24.3	73.4	1282.24	17.53	92.50	2.11	0.11	27.9	70.9	1034.37
3	34.18	96.90	233	1.78	33.7	267.5	274.05	20.82	68.00	21.5	0.57	30.3	147.1	421.45
4	18.23	95.90	116	0.92	24.2	71.6	440. 58	10.12	73.10	7.36	0.43	25.4	67.2	374.28
5	10.60	91.1	306	0.53	24.5	74.3	844.53	6.52	72.70	41.30	0.11	26.1	70.8	804.42
6	16.52	93.4	10.4	0.44	24.2	46.6	438.35	6.61	67.90	4.23	0.12	28.8	72.3	477.85
7	15.63	93.2	66.90	0.089	30.2	36.1	823.34	3.95	48.70	2.15	0.14	35.7	46.9	318.36

*Abbreviation*: *WBC* White blood cell, *N%* Percentage of neutrophils, *CRP* C reactive protein, *PCT* Procalcitonin, *ALB* Serum albumin, *Sr* Serum creatinine, *24 h UP* 24-h urinary protein

*: *P* < 0.05, compared to data before treatment; ^a^: All the data in “after treatment” were obtained when antibiotic treatment stopped

**Table 3:** The clinical manifestations of 7 patients affected by Nocardia

Patient	Hematuria	Clinical manifestations Of respiratory system	Fever	Position of abscess
1	–	Cough, expectoration	38.8 °C	Disseminated (Right lower abdomen, left hip, right brain)
2	+	Cough, expectoration	–	Right thigh
3	–	Dyspnea, chest distress, pleural effusion	–	Left thigh, lung
4	–	–	–	Right middle finger
5	–	Cough, expectoration	39.5 °C	Right neck, lung
6	+	Pleural effusion	–	Neck, lung, right hip
7	+++	Cough, dyspnea	40 °C	Disseminated (Front chest, back, limbs, lung, brain)

### Response to the treatment of antibacterial therapy

All 7 patients accepted antibiotic treatment once they were diagnosed. One patient received a single regimen with compound sulfamethoxazole, and 5 patients were treated with a double-regimen antibiotic treatment in which compound sulfamethoxazole was prescribed with a combination of linezolid, imipenem and mezlocillin/sulbactam for 2 patients, 2 patients and 1 patient, respectively (Table 4). Meanwhile, 1 serious disseminated Nocardiosis patient was performed intracranial medication as well as subcutaneous abscess puncture drainage and three kinds of antibiotics; and other 6 patients did not undergo abscess puncture drainage. Encouragingly, after antibiotic treatment and abscess drainage, abscess diminished or disappeared ranging from 3 days to 80 days. By CT and MRI, it was shown that pulmonary and cerebral abscess also diminished as well. Moreover, inflammatory biomarkers, including neutrophilia count, CRP and PCT significantly declined (*p*<0.05) compared to that of before treatment; proteinuria excretion slightly ameliorated (Table 2). When abscess disappeared and inflammatory indicators improved, antibiotic treatment was stopped. The duration of antibiotic treatment and follow-up for these patients ranging from 3 months to 6 months (Table 5).

**Table 4:** The category and course of antibiotics treatment in 7 patients

Patient	Usage of antibiotics	
	Category	Course
1	Compound sulfamethoxazole, Imipenem	4.5 months
2	Compound sulfamethoxazole	3 months
3	Compound sulfamethoxazole, sulfadiazine sodium, Imipenem	3.5 months
4	Compound sulfamethoxazole, Mezlocillin/Sulbactam	loss to follow-up
5	Compound sulfamethoxazole, Linezolid	3 months
6	Compound sulfamethoxazole, Linezolid	3 months
7	Compound sulfamethoxazole, Linezolid, Imipenem	6 months

**Table 5:** The time interval of mass begins reducing after antibiotics treatment and length of follow-up among 7 patients

Patient	Size of abscess		Time to abscess reduction	Length of follow-up
	Before treatment	After treatment		
1	brain: 10 × 8 mm	brain: decrease	buttock: 8 days	4.5 months
	buttock: 141 × 43 mm	buttock: decreased	intracranial: 30 days	
2	–	–	right thigh: 18 days	3 months
3	lung:31 × 36 mm	lung: decreased	lung: 12 days	3.5 months
4	–	–	right middle finger: 3 days	loss to follow-up
5	right neck:74x36mm	right neck: 75x29cm	right neck: 10 days	3 months
	lung:58x68mm	lung: 43x60mm	lung: 10 days	
6	right hip: 60 × 27 mm	right hip: 8 mm in the widest position	neck: 5 days	3 months
			right hip: 17 days	
7	lung: 44x40x41mm	lung: decreased	back: 15 days	6 months
	brain: 10x10mm	brain: 7x4mm	lung: 80 days	
			brain: 2 months	

## Discussion

Nocardiosis is an uncommon infection and is constantly neglected in the clinic in glomerular disease patients. Eleven sporadic glomerular disease cases with nocardiosis have ever been reported in the literature. All these eleven patients accepted immunosuppressive agents for kidney disease; pulmonary infection was the most common; 45% of patients accepted trimethoprim-sulfamethoxazole treatment; and only two patients died [31–34]. Here, we comprehensively reviewed the manifestations of and treatments for nocardiosis in 7 glomerular disease patients who received glucocorticoids alone or combined with immunosuppressive agents. It was delineated that in addition to fever, subcutaneous and pulmonary nodules were major extrarenal manifestations, while renal involvement was nonspecific. Serious nocardiosis cases manifested as dissemination of multisite subcutaneous abscesses concomitant with pulmonary or cerebral abscesses. Compound sulfamethoxazole was effective for nocardiosis in glomerular disease patients and even for disseminated nocardiosis, which was characterized by diminished subcutaneous and pulmonary abscesses as a therapeutic response.

It has been suggested that nocardiosis mainly occurs in patients suffering from autoimmune diseases, chronic renal disease, hematological diseases and HIV infection, especially in those who receive glucocorticoids or other immunosuppressive treatments [29, 35–38]. Here, we observed that all 7 patients with nocardiosis had been receiving glucocorticoids alone or combined with immunosuppressive agents, which is consistent with the previous literature. The diagnosis of nocardiosis mainly includes gram stains, microbial cultures and gene sequencing of pus or serum [39, 40]. The 7 cases of nocardiosis in our center were confirmed through abscess puncture and pus microbial culture. In addition, supplementary examinations showed elevated levels of inflammatory biomarkers, and subcutaneous, pulmonary and cerebral abscesses were detected by CT/MRI.

It has been found that approximately 80% of nocardiosis patients have hypoimmunity through CD4 + Th/Ts counting detection, and glucocorticoids are the crucial risk factor for nocardiosis development [41]. We observed that the median dosage of methylprednisolone was 20 mg per day, and the median use time was 4 months before nocardiosis diagnosis. Six patients received methylprednisolone for or more than 4 months, and 4 patients had low lymphocyte counts, which revealed that long-term steroid treatment might increase the risk of Nocardia infection.

The clinical manifestations are very complicated and difficult to distinguish promptly in the clinic. Hematuria, proteinuria, serum albumin levels and serum creatinine levels did not show significant differences before or after nocardiosis, which indicates that the inflammation associated with nocardiosis did not worsen the glomerular disease in our 7 patients. It was observed that extrarenal presentations included fever, cough, and multisite abscesses. Only 3 out of 7 patients had fever, so fever is not always present in nocardiosis patients. Multisite abscesses includes subcutaneous, pulmonary and cerebral abscesses [42, 43], which are commonly misdiagnosed as pneumonia or tuberculosis or even as lung cancer or metastatic tumors. Considering that direct extrinsic contact and inhalation are the main routes of Nocardia infection, subcutaneous and lung tissues are the most frequently affected sites of nocardiosis [44, 45]. Here, it was observed that all 7 nocardiosis patients presented with subcutaneous abscesses, and 4 of them presented with concomitant pulmonary abscesses, suggesting that subcutaneous and pulmonary abscesses are important signs of nocardiosis in glomerular disease patients who receive glucocorticoids alone or combined with immunosuppressive therapy. In addition, disseminated nocardiosis is delineated to spread to the brain with or without central nervous system (CNS) manifestations [24]. We also found that 2 nocardiosis patients with cerebral abscesses exhibited no CNS symptoms, indicating that CT/MRI is indispensable for nocardiosis patients, especially in patients with disseminated nocardiosis.

*Nocardia spp*. are susceptible to various antibiotics, including sulfonamide, linezolid, imipenem and amikacin. Among these, sulfonamide is recommended as the optimal therapy [46–48]. In addition, 94.6% of Nocardia isolates are susceptible to trimethoprim-sulfamethoxazole [49]. Therefore, the 7 nocardiosis patients in our center were treated with sulfonamide alone or in combination with other antibiotics for 3 months to 6 months, and no relapse was found during the follow-up. Moreover, previous literature has suggested that long-term antibiotic treatment for more than 3 months, even 6–12 months, might be beneficial for low nocardiosis relapse rates [50, 51]. Thus, the appropriate duration of sulfonamide for nocardiosis in glomerular disease needs to be further clarified. In our center, cerebral abscesses in 2 disseminated nocardiosis patients obviously decreased in size after combination treatment with sulfonamide and linezolid or imipenem. For some nocardiosis patients with cerebral abscesses, surgery is necessary in addition to antibiotic therapy [52].

## Conclusion

In summary, multisite abscesses, including subcutaneous, pulmonary and cerebral abscesses, with or without fever, are the common manifestations of nocardiosis in glomerular disease patients with glucocorticoid and immunosuppressive therapy. Sulfonamide was the first-line antibiotic treatment for nocardiosis, and combinations of other antibiotics are also needed in some serious cases.

## Data Availability

Data sharing is not applicable to this article as no datasets were generated or analysed during the current study.

## Abbreviations

ESRD: End-stage renal disease
CRP: C-reactive protein
PCT: Procalcitonin
CT: Computed tomography
MRI: Magnetic resonance imaging
eGFR: Estimated glomerular filtration rate
AKI: Acute kidney injury
HIV: Human immunodeficiency virus
CNS: Central nervous system
